# Changes in Parent and Child Skin Carotenoids, Weight, and Dietary Behaviors over Parental Weight Management

**DOI:** 10.3390/nu13072227

**Published:** 2021-06-29

**Authors:** Keeley J. Pratt, Emily B. Hill, Haley M. Kiser, Catherine E. VanFossen, Ashlea Braun, Christopher A. Taylor, Colleen Spees

**Affiliations:** 1 Human Development and Family Science Program, Department of Human Sciences, College of Education and Human Ecology, The Ohio State University, Columbus, OH 43210, USA; kiser.105@osu.edu (H.M.K.); cav79@drexel.edu (C.E.V.); 2 The Ohio State Wexner Medical Center, Department of Surgery, Columbus, OH 43210, USA; 3 Department of Pediatrics, The Ohio State University, Columbus, OH 43210, USA; 4 Divison of Medical Dietetics, School of Health and Rehabilitation Sciences, The Ohio State University College of Medicine, Columbus, OH 43210, USA; hill.1224@osu.edu (E.B.H.); braun.165@osu.edu (A.B.); chris.taylor@osumc.edu (C.A.T.); colleen.spees@osumc.edu (C.S.)

**Keywords:** skin carotenoids, parent–child, weight management, fruit and vegetable intake

## Abstract

(1) The objective was to determine changes in parent–child (ages 7–18) dyad skin carotenoids spanning parental participation in a medical weight management program (WMP), and associations with parent BMI, child BMIz, fruit/vegetable intake, and family meals and patterns. (2) The study design was a longitudinal dyadic observational study with assessment at WMP initiation, mid-point (3-months), and conclusion (6-months). Twenty-three dyads initiated the study, 16 provided assessments at 3 months, and 11 at program conclusion. Associations between parent and child carotenoids (dependent variables) and parent BMI, child BMIz, increases in fruit/vegetable intake, and family meals and patterns were analyzed using Pearson’s correlations and independent samples t-tests. Repeated measures ANOVA assessed changes in weight status and carotenoids. (3) Parents experienced significant declines in BMI and skin carotenoid levels over 6 months. Parent and child carotenoids were correlated at each assessment. At initiation, parent BMI and carotenoids were inversely correlated, child carotenoids were associated with increased family meals, and never consuming an evening fast food or restaurant meal were associated with increased parent and child carotenoids. (4) Results demonstrate skin carotenoids are strongly correlated within dyads and may be associated with lower parental BMI and positive family meal practices.

## 1. Introduction

Adult obesity rates have increased over the past three decades [1,2], with half of all United States (US) adults pursuing some form of weight loss, often through medical weight management programs (WMPs) [3,4]. Given that parental obesity is the strongest risk factor for childhood obesity, these children are three-times more likely to develop obesity and future comorbidities extending into adulthood, as well as a shortened life expectancy [5,6,7,8,9,10,11,12]. Yet, we know very little about how parental participation in adult medical WMPs affects children’s dietary behaviors [13]. Despite convincing evidence that parental participation in children’s medical WMPs has positive effects on both child and parent behaviors and weight [14,15,16,17], there is a paucity of data on whether these same trends emerge when parents are the identified patient in these programs, and the child is not the focus. This is an important limitation with significant implications given that 50% of US adults are attempting weight loss many with children living with them who are at risk of obesity. Thus, understanding the influence of parental participation in medical WMPs on children’s dietary behaviors is essential information needed before targeted interventions can be designed to reach a subgroup of parent–child dyads for which there are currently no tested interventions, and for children at high risk of having obesity.

Prior research conducted with children of parents in adult medical WMPs is predominately cross-sectional [13,18,19,20,21], retrospective [22,23], and isolated to parent-only perspectives [13,18,20]. Prospective assessments of dyads’ objectively measured dietary behaviors are needed to determine short- and long-term effects of parental participation on children. Given that fruit and vegetable intake is part of a healthy diet and is a target in WMPs, finding accurate and feasible ways to track fruit and vegetable intake over WMP is necessary. However, obtaining parent and child objective measures of diet is time- and resource-intensive. One innovative, non-invasive way is through validated resonance Raman spectroscopy to measure carotenoid status in skin as a biomarker of fruit/vegetable intake [24,25]. Carotenoids are phytochemicals present in many fruits and vegetables that are distributed in human tissues after consumption. Assessment of carotenoid status has been validated for use as an indicator of dietary intake [26]. Objective measures of skin carotenoids consistently demonstrate moderate to strong correlations with total serum or plasma carotenoids, and are positively associated with intake of fruits and vegetables as assessed by food frequency questionnaires and automated multiple-pass 24-h daily recalls among children ≥5 years-old [26,27,28,29]. This method accounts for greater accuracy in reporting, especially from children, who often provide subjective reports for dietary intake [26,27,28,29].

The objective of the study was to determine changes in parent and child fruit and vegetable intake via skin carotenoids and changes in weight (BMI, BMIz) over parental participation in an adult outpatient medical WMP, and associations between parent and child carotenoids and changes in weight, reported fruit and vegetable intake, and family meal frequency and patterns. It was hypothesized that: (1) parents experience significant decreases in BMI and increases in skin carotenoids; (2) parent and child skin carotenoids are correlated at each visit; (3) higher skin carotenoids in parents and children are associated with lower parent BMI and child BMIz at each visit; (4) higher parent and child skin carotenoids are associated with parent and child reports of increased fruit and vegetable intake at each visit; (5) more frequent family meals are associated with higher parent and child skin carotenoids at each visit; and (6) more frequent fast food, delivery, and restaurant/carry out consumption are associated with lower parent and child skin carotenoids at each visit.

## 2. Materials and Methods

### 2.1. Study Design and Participants

The study was a part of a larger study about the effects on children from parental participation in an adult medical WMP. The single group, longitudinal design utilized objective and survey assessments from WMP initiation, 3-months (mid-program), and 6-months (program end). Recruitment took place at parents’ WMP orientation from November 2018 to March 2020, in which every eligible parent–child dyad was invited to participate. Details about the WMP at The Ohio State University Comprehensive Center for Weight Management, Metabolic and Bariatric Surgery have been described previously [30,31]. In short, the 6-month program includes nutrition, exercise, and behavioral components delivered through group educational and support classes and individual consultations with registered dietitians, behavioral health providers, and exercise physiologists. As part of the nutrition education, fruit and vegetable intake is encouraged throughout classes and consultations. There is an initial wellness orientation, in which patients have their resting metabolic rate tested and a fitness evaluation to assist in formulating individualized meal and exercise plans, respectively. Weight, dietary, and exercise journals are reviewed weekly with a post-program fitness evaluation.

Inclusion criteria comprised: parent enrolled in the WMP, child aged 7–18 years old living in the home ≥4 days per week with the parent, no parent or child history of bariatric surgeries, no life-threatening comorbidities, and free of conditions that would prevent engagement in physical activity. The child age range of 7–18 was selected due to appropriateness of child self-report measures. If multiple children met inclusion criteria, parents were encouraged to select their youngest child. Parents who indicated interest in participation in this study at their wellness orientation were provided contact information to schedule with their child, in which consent/assent were obtained and dyads were enrolled.

### 2.2. Procedures

Parents and children had measurements of height, weight, and skin carotenoids taken, and completed a research packet that contained the measures described below the week the parent began the WMP, 3 months into the program (the half-way point), and at 6 months upon program conclusion. Parents and children had assessments completed at the University Hospital where the WMP is located. There were several exceptions when after school scheduling became challenging, where children had their measures and assessments completed with a member of the research team traveling to their home. Children were offered support from a member of the research team if they needed assistance with completing the measures. Parents and children each received a $20 retail gift card for participation at each assessment. All subjects provided informed consent before participating in the study. The study received Institutional Review Board approval.

### 2.3. Measures

Skin carotenoids were measured by trained research staff from parents and children using a Pharmanex NuSkin BioPhotonic S3 Scanner (NuSkin Enterprises, Provo, UT, USA). This scanner uses resonance Raman spectroscopy to estimate skin carotenoids and is correlated with fruit and vegetable intake [32,33]. Parents and children completed three consecutive readings on the palm of the hand. The average of the three readings was used at each time point.

Parent and child weight status was calculated based on measured height and weight using a wall-mounted stadiometer and a scale [34]. Parent BMI was calculated, and using child date of birth, child BMI, child BMI percentile, and child BMI z-score (BMIz) were calculated, and weight status categories were determined. Child BMI percentile is used to account for changes in body composition as children age and based on sex, using age and sex specific percentiles for BMI rather than raw BMI which is used for adults. Child BMI z-score is a measure of BMI adjusted for child age and sex on a standard growth chart, and is the standard way to measure changes for children of diverse weight status (i.e., under, healthy, overweight, obese).

Increases in fruit and vegetable intake was self-reported from parents and children using one question from Project EAT [35]: “Have you increased your fruit and vegetable intake in order to lose weight or to keep from gaining weight in the past 30 days?” Dichotomous response options include yes or no. Project EAT questions have been pilot tested and have a test–retest agreement of 88% for healthy behaviors, including the question used in this study [35].

Family meal practices were assessed from parents using four questions from the Project EAT survey [35]: “During the past 7 days, how many times did all, or most of your family living in your home eat dinner or supper together?” “During the past 7 days was a family evening meal purchased from a fast-food restaurant and eaten at the restaurant or at home?” “… was an evening meal delivered to your home?” “… was a family evening meal picked up as takeout food?” These questions have been published with good reliability [36,37]. Questions were answered on a Likert scale: Never (0), 1–2, 3–4, 5–6, and 7 times per week. Researchers have scored the questions in different ways, using the individual family meals practices primarily as a categorical variable or dichotomizing responses per week [38,39,40]. For our analysis, and based on the distribution of participant responses in which there was distribution across the Likert options, “eating dinner or supper together” was dichotomized into “1–4 times per week” and “more than 4 times.” The other questions about evening meals being purchased from “fast food restaurants,” “food delivery,” and “food carry out” were dichotomized into “never” and “at least one time per week” since there was limited variance in participant responses across the Likert options. These questions have good reliability [35,37].

### 2.4. Analysis

Initial analysis included descriptive and bi-variate statistics. Descriptives were conducted for all variables of interest: parent and child skin carotenoids, parent BMI, child BMIz, parent and child reports of increased fruit/vegetable intake, parent reports of family meals. To test hypotheses 1, repeated measures ANOVA was conducted using a mixed multilevel modeling approach to estimate values using Restricted Maximum Likelihood (REML), which is the ideal estimation method for missing values accounting for smaller sample sizes [41]. There were four models conducted with primary parent and child outcome variables in the time effect models including (1) parent fruit and vegetable intake via skin carotenoids, (2) child fruit and vegetable intake via skin carotenoids, (3) parent BMI, and (4) child BMIz. The models incorporated data from all three-assessment points to determine the time effects. Because of the small sample size and attrition, fixed factors and covariates were not included in the models. To test hypotheses 2–3, Pearson’s correlations were run between child and parent skin carotenoids and BMI/BMIz at each time point (i.e., baseline, 3-month, 6-month). To test hypotheses 4–6, independent samples t-tests were conducted with each independent categorical variable (parent and child increased fruit/vegetable intake, family evening meal together, fast food, delivery, and carry-out) and dependent variable (parent and child skin carotenoids and BMI/BMIz) at each of the three assessment time points. Analysis was conducted using SPSS version 27 (IBM Corp., Armonk, NY, USA). Significance was set at *p* < 0.05.

## 3. Results

### 3.1. Sample and Demographics

There were 53 parents with children meeting inclusion criteria invited to participate in the study. Twenty-three (43.3%) dyads provided consent/assent and completed the first assessment the week the parent started the medical WMP. Thirty dyads did not provide consent due to not returning initial contact, lack of interest from child, or time constraints. At the 3-month (mid-program) assessment, 16 dyads completed the assessment and skin carotenoids. At the 6-month (end of program assessment), 11 dyads completed the assessment and skin carotenoids. There were no significant demographic differences between those that continued in the study and those that dropped out. Of those that dropped out of the study at 3-months, 2 dropped out of the WMP, 4 did not want to continue in the study (3 no response, 1 perceived child as uninterested), and due to COVID-19 restrictions 1 could not have their skin carotenoids obtained. At 6-months, 2 did not want to continue in the study (no response, stated no time), 1 dropped out of the WMP, and due to COVID-19 restrictions 2 could not have their skin carotenoids obtained.

Parent and child demographics are provided in Table 1. Parents were an average age of 43 and identified as predominately female (78%), White (74%), non-Hispanic (100%), married (70%), with class II or III obesity (87%), and with at least a Bachelor’s degree (87%). On average, parents had two children living in the home. Children were an average age of 12 and were equally split in sex (52% female). The majority of children identified as White (65%), non-Hispanic (83%), and were divided between a healthy weight (52%) and overweight/obese weight status (48%).

### 3.2. Descriptive Statistics

The descriptive statistics for all variables at each time point are provided in Table 2. Parents had very high reports of increased fruit/vegetable intake at program initiation, 3 months, and 6 months (96%, 94%, 82%); while children had more modest reports at these time points (35%, 50%, 36%). Over half (57%) of parents reported eating family dinner/supper together more ≥4 times per week at program initiation, which decreased slightly at 3 months (44%) and increased at 6 months (73%). Parents’ reports of eating evening meals that were fast food (70%, 69%, 73%), delivered (52%, 44%, 64%), or at a restaurant or take out (48%, 50%, 55%) remained largely unchanged from program initiation through 6 months. There were no statistically significant differences between the measures in Table 2 over the three visits.

### 3.3. Hypotheses

Hypothesis 1 was partially supported in which parents experienced significant declines in BMI [F(2, 25.9) = 6.9, *p* = 0.004] with a small effect size (η^2^ = 0.019) over 6 months (see Table 3). However, parents experienced significant decreases in skin carotenoids over six months [F(2, 25.9) = 4.34, *p* = 0.024] with a small (η^2^ = 0.019) effect size. There were no significant time effects for child BMIz or child skin carotenoids, though there was a small effect size for child BMIz (η^2^ = 0.015).

Hypothesis 2 was partially supported in which parent and child skin carotenoids were positively correlated at each time point [program initiation: r(21) = 0.65, *p* < 0.001; 3-month: r(14) = 0.50, *p* = 0.05; and 6-month: r(11) = 0.70, *p* = 0.02].

Hypothesis 3 was partially supported, in which parent BMI was inversely correlated with parent skin carotenoids at program initiation only, r(21) = −0.44, *p* = 0.03. Although not statistically significant, for both parents and children, lower BMI/BMIz was associated with higher skin carotenoids at each time point (see Table S1).

Hypothesis 4 was not supported, in which there were no significant differences in child skin carotenoids between those with and without reported increases in fruit/vegetable consumption at program initiation (see Table 4). Although not statistically significant, children who reported increased fruit/vegetable consumption had higher skin carotenoids at each time point. We could not assess for differences between groups based on parent responses due to the high number of parents who reported increased fruit/vegetable consumption (96%).

Hypothesis 5 was partially supported, in which child skin carotenoids at program initiation were higher for those participating in a greater number of family dinners (3–7 per week) (see Table 4). Although not significant, children and parents with a greater number of family dinners per week (more than 4 times) had higher skin carotenoids at each time point.

Hypothesis 6 was also partially supported, in which never consuming a weekly evening fast food meal was significantly associated with higher child and parent skin carotenoids at program initiation (see Table 4). Additionally, never eating an evening weekly meal at/or carry out from a restaurant was significantly associated with higher child skin carotenoids at program initiation; there were no significant differences in skin carotenoids based on restaurant meal consumption for parents or based on delivery of meals for either parents or children.

## 4. Discussion

The prospective, longitudinal design of this study allows for novel insight into parent and child changes in skin carotenoids and weight during parental participation in an adult medical WMP. Importantly, this is the first study to examine the relationship between parental participation in a WMP and objective indicators of fruit and vegetable consumption in parent–child dyads. These results build upon our previous work with parents in WMPs, and demonstrate parent and child dietary behaviors, including intakes of fruits and vegetables, are positively correlated [20]. Results also showed parental weight loss as a result of the WMP, and decreases in parental skin carotenoid levels. This provides preliminary evidence of how parental participation in WMPs affects children’s dietary behaviors, and identifies a need for future investigation into sustained effects for both parents and children.

Despite parental weight loss, in contrast to expectations, we observed decreases in parent skin carotenoids over the course of WMP participation. However, nearly all parents in the study reported increases in fruit and vegetable intakes at each time point, including initiation. This suggests parents may have increased consumption of fruits and vegetables in an effort to lose weight prior to WMP initiation, and therefore change in consumption of these foods was not a dietary strategy employed throughout WMP participation. Parents may have focused instead on other weight management tactics, such as portion control or energy restriction which were not assessed in this study. As a result, the observed decrease in skin carotenoids may be related to an overall reduction in intakes as part of a goal to lower energy intake. It is also plausible this strategy led to a marked decrease in consumption of energy-dense, fat-containing foods, which are necessary for the absorption and distribution of lipid-soluble carotenoids [42]. The absence of significant change in child skin carotenoids throughout parental participation in WMP may also be explained by these factors, as parent focus on reductions in total energy intake, rather than increases in fruit and vegetable consumption, may not have led to differences in the home environment related to fruit and vegetable availability and meal composition and, therefore, would not be expected to affect fruit and vegetable intakes among children [43].

Relationships between skin carotenoids and family meal practices among participants in this study provide preliminary objective evidence for the impact of these behaviors on fruit and vegetable intakes within the context of parental WMPs. Increased frequency of family meals was positively correlated with child skin carotenoids at program initiation. Further, trends suggest an association between greater number of family meals and higher skin carotenoids among both children and parents for all time points throughout WMP participation. This indicates these meals serve as an important source of fruits and vegetables for children and parents. This observation is supported by previous literature, which has demonstrated positive relationships between family meal frequency and consumption of fruits and vegetables as well as associations with greater overall diet quality [44]. Our findings suggest fruits and vegetables are regularly consumed as part of family meals, even if relationships between skin carotenoids and increases in consumption of fruits and vegetables as part of the parental WMP were not identified.

Higher skin carotenoids were associated with lower frequency of consumption of meals prepared away from home, with significant inverse relationships identified for parents and children for fast food consumption and for children with restaurant or carry out meals at WMP initiation. These results suggest meals purchased outside the home are lower in fruits and vegetables than those prepared within the home. Previous literature also demonstrated this relationship, with away from home meals associated with poorer diet quality and lower fruit and vegetable intakes among dyads [45]. The lack of a significant relationship between skin carotenoids and meals away from home at 3- and 6-months, may indicate improvements in dietary behaviors as a result of strategies learned through parental participation in WMP.

Though this study has many strengths, including longitudinal data collection, a well-established adult WMP, enrollment of parent–child dyads, and objective indicators of fruit and vegetable consumption and height/weight, it is not without limitations. As a single-arm study, the lack of a control group limits the ability to determine the causal effect of the WMP on outcomes. Further, the small sample size, attrition, and homogeneity of participants as well as the use of a convenience sample may limit generalizability to other populations participating in adult weight management settings. The way that fruit and vegetable consumption was measured included combined increases in “fruit and vegetable” intake, and did not include the preparation of these foods (i.e., raw, processed, etc.). Bioavailability of carotenoids varies based upon the food matrix, processing conditions, preparation, and several other factors, lack of information on these details in this study impedes the ability to fully elucidate mechanisms that may have impacted changes in skin carotenoids over the course of the WMP, and thus results should be interpreted with caution [46]. Finally, future work would benefit from including assessments of 24-h food recalls or food frequency questionnaires to confer with parental and child skin carotenoids.

## Figures and Tables

**Table 1:** Parent and Child Demographics (N = 23) [%(n) or Mean ± SD].

Parent		Child	
Female		Female	
Yes	78.3 (18)	Yes	52.2 (12)
Race		Race	
White	73.9 (17)	White	65.2 (15)
African American/Black	17.4 (4)	African American/Black	17.4 (4)
Asian	8.7 (2)	Multiracial	8.7 (2)
		Other	8.7 (2)
Hispanic		Ethnicity	
Yes	0	Yes	17.4 (4)
Age (years)	43.4 ± 5.74	Age (years)	12.3 ± 3.27
Weight Status		BMI	23.2 ± 6.51
Class I Obesity	13.0 (3)	BMI Percentile	73.0 ± 27.2
Class II Obesity	34.8 (8)	Weight Status	
Class III Obesity	52.2 (12)	Healthy Weight	52.2 (12)
Education		Overweight	17.4 (4)
High School Graduate	4.35 (1)	Obese	30.4 (7)
Associate Degree	8.70 (2)		
Bachelor’s Degree	69.6 (16)		
≥Master’s Degree	17.4 (4)		
**Household**			
Annual Household Income		Parental Relationship Status	
$40,000–59,999	17.4 (4)	Married	69.6 (16)
$60,000–99,000	34.8 (8)	Divorced	17.4 (4)
$100,000+	47.8 (11)	Single	8.70 (2)
Number of Children	2.00 ± 1.13		

**Table 2:** Parent and Child Average Scores at Baseline, 3 months, and 6 months.

	Baseline (*n* = 23)	3 Months (*n* = 16)	6 Months (*n* = 11)	*F*(df) or *x*^2^ (df, N)	*p* Value
Parent carotenoids (RRS)	26,699 ± 10,823	28,715 ± 12,496	24,595 ± 9355	0.455 (2, 47)	0.637
Child carotenoids (RRS)	28,234 ± 12,752	29,901.65 ± 14,338	30,637 ± 13,336	0.134 (2, 27)	0.875
Parent BMI (kg/m^2^)	43.7 ± 8.68	42.9 ± 9.13	41.9 ± 8.53	0.449 (2, 47)	0.641
Child BMIz	0.97 ± 1.18	0.93 ± 1.18	0.83 ± 1.26	0.361 (2, 47)	0.699
Parent increased fruit and vegetable intake (% yes)	22 (95.7%)	15 (93.8%)	9 (81.8%)	2.03 (2, 50)	0.362
Child increased fruit and vegetable intake (% yes)	8 (34.8%)	8 (50.0%)	4 (36.4%)	0.988 (2, 50)	0.610
Family dinner/supper (% >4 times per week)	13 (56.5%)	7 (43.8%)	8 (72.7%)	2.23 (2, 50)	0.329
Evening meal fast-food (% at least 1 per week)	16 (69.6%)	11 (68.8%)	8 (72.7%)	0.053 (2, 50)	0.974
Evening meal delivery (% at least 1 per week)	12 (52.2%)	7 (43.8%)	7 (63.6%)	1.03 (2, 50)	0.597
Evening meal restaurant or takeout (% at least 1 per week)	11 (47.8%)	8 (50.0%)	6 (54.6%)	0.134 (2, 50)	0.935

**Table 3:** Results of Repeated Measures ANOVA for Child and Parent Skin Carotenoid and BMI/BMIz.

	Child-Reports			Parent-Reports		
	df	F	η^2^	df	F	η^2^
Skin Carotenoid	2, 27.2	0.60	0.006	2, 25.9	4.34 *	0.019
BMIz and BMI	2, 25.2	0.09	0.015	2, 25.3	6.85 **	0.019

* *p* < *0*.05, ** *p* < 0.01. Eta-squared η^2^ = 0.01 small effect; 0.06 = medium effect; 0.14 = large effect.

**Table 4:** Independent t-tests Based on Child Fruit and Vegetable Intake, Family Dinner, and Evening Meals.

	Baseline (Program Start; *n* = 23)			3 Months (Program Midpoint; *n* = 16)			6 Months (Program End; *n* = 11)		
	Mean ± SD	t (df)	*p*	Mean ± SD	t (df)	*p*	Mean ± SD	t (df)	*p*
**Child Fruit/Vegetable Increase**									
Child carotenoids (RRS)									
Yes	33,075.92± 16,252.72	−1.25 (21)	0.25	31,449.75 ± 10,167.68	−1.95 (14)	0.68	34,758.92 ± 22,094.63	−0.58 (3.25)	0.60
No	25,651.78 ± 12,024.15			28,353.54 ± 18,212.24			28,281.95 ± 5877.64		
**Family Dinner-Eat Together**									
Child carotenoids (RRS)									
1–4 times	21,080 ± 10,744	−2.42 (21)	0.03 *	26,424.40 ± 12,991.91	−1.11 (14)	0.29	27,105.55 ± 8644.07	−0.52 (9)	0.62
5–7 times	33,738 ± 13,590			34,372.38 ± 15,730.89			31,961.58 ± 15,012.81		
Parent carotenoids (RRS)									
1–4 times	23,989 ± 8929	−1.06 (21)	0.30	25,587.00 ± 9708.34	−1.15 (14)	0.27	21,641.44 ± 1286.63	−0.62 (9)	0.55
5–7 times	28,783 ± 12,005			32,735.48 ± 15,204.05			25,701.25 ± 10,927.38		
**Dinner-Fast-food**									
Child carotenoids (RRS)									
Never	38,129 ± 16,548	2.55 (21)	0.02 *	33,575.60 ± 10,266.96	0.68 (14)	0.51	41,715.33 ± 21,934.81	1.30 (2.10)	0.32
At least once	23,906 ± 10,108			28,231.67 ± 16,012.52			26,104.17 ± 5525.37		
Parent carotenoids (RRS)									
Never	34,251 ± 12,780	2.45 (21)	0.02 *	24,331.87 ± 12,804.74	−0.94 (14)	0.36	30,929.44 ± 15,075.78	1.45 (9)	0.18
At least once	233,394 ± 8258			30,706.55 ± 12,000.97			22,218.25 ± 6035.60		
**Dinner–At Restaurant or Carry Out**									
Child carotenoids (RRS)									
Never	33,177 ± 7515	1.91 (21)	0.07	25,630.21 ± 11,238.01	−0.98 (14)	0.34	27,609.07 ± 4950.52	−0.67 (9)	.52
At least once	22,843 ± 17,107			31,798.71 ± 12,658.86			33,160.67 ± 17,868.94		
Parent carotenoids (RRS)									
Never	28,242 ± 9453	0.71 (21)	0.49	30,813.42 ± 13,473.99	0.25 (14)	0.81	22,029.93 ± 5140.29	−0.82 (9)	0.44
At least once	25,015 ± 12,385			28,989.88 ± 16,034.24			26,730.78 ± 11,909.38		
**Dinner-Delivery**									
Child carotenoids (RRS)									
Never	32,684 ± 15,054	1.53 (21)	0.14	33,020.19 ± 15,327.51	0.98 (14)	0.34	26,124.08 ± 5053.05	−0.84 (9)	0.43
At least once	24,155 ± 11,588			25,892.10 ± 12,937.63			33,126.14 ± 16,196.46		
Parent carotenoids (RRS)									
Never	27,169 ± 11,502	0.20 (21)	0.85	29,026.15 ± 13,096.99	0.11 (14)	0.91	22,246.92 ± 5692.56	−0.61 (9)	0.56
At least once	26,268 ± 10,657			28,313.71 ± 12,701.42			25,935.24 ± 11,130.15		

* *p* < 0.05.

## Supplementary Materials

The following are available online at https://www.mdpi.com/article/10.3390/nu13072227/s1, Table S1: Correlation Matrix between Child and Parent Weight Status and Skin Carotenoids at Baseline, 3-months, and 6-months.
