# Supplementary material for: Changes in Parent and Child Skin Carotenoids, Weight, and Dietary Behaviors over Parental Weight Management
Source: Nutrients. 2021 Jun 29;13(7):2227. doi: 10.3390/nu13072227 (PMC8308330; doi:10.3390/nu13072227)
Supplement: Supplementary file 1 [file nutrients-13-02227-s001.zip › nutrients-1278729-supplementary.pdf]

Supplemental Table S1:
Correlation Matrix between Child and Parent Weight Status and Skin Carotenoids at Baseline, 3-months, and 6-months

	Child BMIz	Parent BMI	Child Skin Carotenoids	Parent Skin Carotenoids
Baseline (N=23)				
Child BMIz	-			
Parent BMI	r=.450*<br>p=.031	-		
Child Skin Carotenoids	r=-.286<br>p=.186	r=-.309<br>p=.152	-	
Parent Skin Carotenoids	r=-.389<br>p=.066	r=-.443*<br>p=.034	r=.651**<br>p=.001	-
3-months (N=16)				
Child BMIz	-			
Parent BMI	r=.691**<br>p=.003	-		
Child Skin Carotenoids	r=-.212<br>p=.430	r=-.304<br>p=.253	-	
Parent Skin Carotenoids	r=-.205<br>p=.447	r=-.373<br>p=.154	r=.499*<br>p=.049	-
6-months (N=11)				
Child BMIz	-			
Parent BMI	r=.729*<br>p=.011	-		
Child Skin Carotenoids	r=-.165<br>p=.627	r=-.117<br>p=.731	-	
Parent Skin Carotenoids	r=-.346<br>p=.298	r=-.178<br>p=.600	r=.699*<br>p=.017	-

**Correlation is significant at the .01 level; *Correlation is significant at the .05 level
